# Hepatobiliary Scintigraphy and Glass ^90^Y Radioembolization with Personalized Dosimetry: Dynamic Changes in Treated and Nontreated Liver

**DOI:** 10.3390/diagnostics11060931

**Published:** 2021-05-21

**Authors:** Carole Allimant, Emmanuel Deshayes, Marilyne Kafrouni, Lore Santoro, Delphine de Verbizier, Marjolaine Fourcade, Christophe Cassinotto, Margaux Hermida, Chloé Guillot, Denis Mariano-Goulart, Boris Guiu

**Affiliations:** 1 Department of Radiology, St-Eloi University Hospital, 34000 Montpellier, France; c-allimant@chu-montpellier.fr (C.A.); c-cassinotto@chu-montpellier.fr (C.C.); m-hermida@chu-montpellier.fr (M.H.); chloe-guillot@chu-montpellier.fr (C.G.); 2 Department of Nuclear Medicine, Institut du Cancer Montpellier, 34000 Montpellier, France; emmanuel.deshayes@icm.unicancer.fr (E.D.); lore.santoro@icm.unicancer.fr (L.S.); 3 Department of Nuclear Medicine, University Hospital Montpellier, 34000 Montpellier, France; marilyne.kafrouni@gmail.com (M.K.); d-de_verbizier@chu-montpellier.Fr (D.d.V.); m-fourcade@chu-montpellier.fr (M.F.); d-mariano_goulart@chu-montpellier.fr (D.M.-G.)

**Keywords:** selective internal radiation therapy, mebrofenin, hepatobiliary scintigraphy, liver

## Abstract

Background: The functional changes that occur over time in the liver following ^90^Y-radioembolization (RE) using personalized dosimetry (PD) remain to be investigated. Methods: November 2016–October 2019: we retrospectively included hepatocellular carcinoma (HCC) patients treated by ^90^Y-glass RE using PD, who underwent hepatobiliary scintigraphy (HBS) at baseline and at 15 days, 1, 2, 3, and 6 months after RE. Results: There were 16 patients with unilobar disease (100%) included, and 64 HBS were performed. Whole liver function significantly decreased over time. The loss was maximal at 2 weeks: −32% (*p* = 0.002) and remained below baseline at 1 (−15%; *p* = 0.002), 2 (−25%; *p* < 0.001), and 3 months (−16%; *p* = 0.027). No radioembolization-induced liver disease was observed. Treated liver function strongly decreased to reach −64% (*p* < 0.001) at 2 months. Nontreated liver function decreased at 2 weeks (−21%; *p* = 0.027) and remained below baseline before reaching +20% (*p* = 0.002) and +59% (*p* < 0.001) at 3 and 6 months, respectively. Volumetric and functional changes exhibited parallel evolutions in the treated livers (*p* = 0.01) but independent evolutions in the nontreated livers (*p* = 0.08). Conclusion: RE using PD induces significant regional changes in liver function over time. As early as 15 days following RE, both the treated and nontreated livers showed a decreased function. Nontreated liver function recovered after 3 months and greatly increased afterwards.

## 1. Introduction

Radioembolization (RE) is a form of brachytherapy in which 90Y microspheres are injected intra-arterially for the internal radiation of liver tumors and, especially, hepatocellular carcinoma (HCC). In intermediate stage HCC (Barcelona Clinic Liver Cancer [BCLC] stage B), time-to-progression (TTP) has been shown to be longer after RE than after transarterial chemoembolization [1,2]. In the BCLC C stage, RE has shown significantly higher response rates and longer TTP than sorafenib (i.e., the standard of care), but phase III trials failed to demonstrate any improvement of overall survival (OS) after RE [3,4,5]. Several hypotheses were raised to explain the negativity of phase III trials, among which dosimetry considerations have recently gained great interest. For radio-induced deterministic effects, a threshold absorbed dose is mandatory to observe an effect [6]. In phase III trials, the activity of ^90^Y to administer was calculated based either on the body surface area with resin 90Y microspheres or on an absorbed dose delivered to the liver (80 to 150 Gy) with ^90^Y glass microspheres [6]. Yet, RE planning should be based on a tumoricidal tumor dose necessary to induce tumor necrosis and on a normal liver dose not to exceed to avoid liver decompensation. A recent post-hoc analysis of one of the negative trials showed increased OS and tumor response in patients with a higher tumor radiation-absorbed dose [7]. The concept of personalized dosimetry, in which the activity of ^90^Y to deliver is based on the pre-RE work-up using [^99m^Tc]Tc-macroaggregate albumin (MAA), emerged in the literature [6,8]. A great step was very recently made with the DOSISPHERE-01 randomized trial, bringing level one evidence to personalized dosimetry with ^90^Y glass microspheres [9].

The main risk of RE is radiation-induced damage to the nontumoral parenchyma, which may cause liver decompensation known as radioembolization-induced liver disease (REILD) [10]. This serious complication is of great importance in HCC, which usually occurs in cirrhotic livers with compromised liver function. In the personalized dosimetry approach, a >30% hepatic reserve (i.e., non-irradiated liver volume) is recommended to limit the risk of REILD [6,9]. However, intuitively, the 30% threshold, which was determined empirically [11], could differ depending on baseline liver function.

Hepatobiliary scintigraphy (HBS), using ^99m^Tc-mebrofenin in combination with SPECT/CT, provides a quantitative evaluation of both the global and regional liver function. HBS has been shown to outperform clinic-biological scores and CT-volumetry in predicting post-hepatectomy liver failure [12,13,14,15,16]. Its ability to monitor regional liver function after RE was evaluated in several reports [17,18,19,20,21] by comparing pre-treatment with one post-treatment HBS. However, functional damage caused by radiation is likely to be a dynamic phenomenon over time, which a single post-treatment HBS evaluation cannot accurately capture. Similarly, the progressive contralateral hypertrophy of the non-irradiated liver [22,23,24] certainly reflects gradual changes in the corresponding liver function.

To our knowledge, no data are available on these dynamic functional changes occurring over time in the treated and nontreated liver parenchyma following RE. This study, therefore, aimed to monitor the changes in regional liver function through repeated HBS examinations in HCC patients treated by RE using personalized dosimetry.

## 2. Materials and Methods

### 2.1. Patient Selection

Among the HCC patients treated by ^90^Y glass radioembolization at our center between November 2016 and October 2019, we retrospectively selected those who underwent sequential evaluations of their liver function using HBS, with a minimum of 2 evaluations, one at baseline and one after treatment. Our indications for RE of HCC were the following: Barcelona Clinic Liver Cancer stage B or C with preserved liver function (i.e., <Child-Pugh B7); unilobar disease with >30% hepatic reserve; Eastern Cooperative Oncology Group stage 0–1; absence of significant extrahepatic disease; normal bilirubin level; and no kidney failure. The RE treatment was validated by our multidisciplinary tumor board after careful review of the work-up phase to eliminate contraindication to RE. Our institutional review board approved this retrospective study (IRB-MTP-2020-01-202000325). Written informed consent was obtained from all patients.

### 2.2. Radioembolization Work-Up

All procedures were performed by experienced interventional radiologists (with >7 years of experience in liver interventional radiology). All patients underwent a pretreatment arteriography to identify the most appropriate location for injection to treat the whole tumor volume, while avoiding injection in the non-tumoral liver parenchyma. [^99m^Tc]Tc-labelled macroaggregated albumin (MAA) was injected as a ^90^Y-microsphere surrogate, and single-photon emission computed tomography (SPECT/CT) images were acquired within one hour after the administration of 99mTc-MAA. SPECT/CT acquisition was performed on a Symbia Intevo 6 SPECT/CT (Siemens Healthcare, Erlangen, Germany) with the following parameters: a window of 140 keV ± 10%, 32 projections per head, 25 s/projection, 128 × 128 matrix, 4.8 mm × 4.8 mm × 4.8 mm voxel size, and a low energy, high-resolution collimator.

SPECT data were reconstructed using a SynbiaNet workstation (Siemens Healthcare) with 5 iterations/8 subsets, attenuation, and scatter corrections using SIEMENS standard commercial solutions. This work-up phase was used to (1) calculate the lung shunt fraction (LSF), (2) to check for complete tumor targeting and for the absence of extrahepatic deposition, and (3) to calculate the activity of the ^90^Y-loaded glass microspheres necessary to treat HCC in accordance with the personalized dosimetry concept [6,9]. The LSF was evaluated using anterior and posterior planar scans and was taken into account to determine the activity to administer. 3D voxel-based dosimetry was performed and evaluated prior to the treatment using a treatment planning system (TPS) (PLANET^®^ Dose, DOSIsoft, Cachan, France) [25,26]. The dosimetry targets were (1) an average absorbed dose of at least 205 Gy to the tumor and more than 250 Gy, if possible; (2) 30 Gy or less to the lungs.

### 2.3. Y90 Microsphere Injection and IMAGING

RE was performed 7–14 days after the work-up phase using Y90-labelled glass microspheres (Theraspheres, BTG International, London, UK) with the microcatheter at the same position as for the MAA work-up. Less than 24 h after RE, a ^90^Y-positron emission tomography (PET)/CT was performed to assess the activity distribution, as previously detailed [25]. The SPECT/CT and ^90^Y-PET/CT were analyzed by a nuclear medicine physician with at least 5 years of experience in RE.

### 2.4. Hepatobiliary Scintigraphy (Morpho-Functional Imaging)

All patients underwent [^99m^Tc]Tc-mebrofenin SPECT-CT imaging using a hybrid scanner (Discovery NM/CT670, GE Healthcare, Milwaukee, Brookfield, WI, USA) at baseline (prior to RE) then at 15 days, 1, 2, 3, and 6 months after RE, according to our follow-up policy over the study period. After an injection of 150 MBq of [^99m^Tc]Tc-mebrofenin (Cholediam, Mediam Pharma, Loos, France), a 6-min dynamic planar acquisition was performed to assess the total liver clearance rate (in %/min/m^2^) normalized to the body surface area. A fast SPECT acquisition was then performed as described elsewhere [27,28]. HBS was performed provided the serum bilirubin level was within a normal range to avoid unreliable evaluation. For both acquisition and post-processing, this technique demonstrated excellent intra- and inter-observer variability [28]. Finally, CT images (2.5 mm slice thickness) were acquired at the portal venous phase using the same system. The Volumetrix^®^ software (GE Healthcare, Milwaukee, Brookfield, WI, USA) was used to reconstruct SPECT data using an iterative algorithm to produce attenuation-corrected images. Co-registration between the CT and SPECT images was visually and manually checked and corrected when required. Regional liver uptake values were determined on the SPECT-CT using the TPS software. We evaluated three different regions of interest (ROIs): the global (whole) liver, the treated liver, and the non-treated liver. A nuclear medicine physician and an experienced radiologist manually segmented the whole liver and treated liver volumes. The treated liver volume was defined by thresholding on 90Y PET/CT images using the TPS. The global, treated, and non-treated liver volumes were transferred to the HBS SPECT/CT using the co-registration matrix previously computed. Manual corrections were applied due to volume changes over time in the three compartments (whole liver, treated, and non-treated liver). The actual [^99m^Tc]Tc-mebrofenin counts in each volume of interest (VOI) were measured and the corresponding regional functions were defined as ((Total counts in the region of interest VOI/Total counts in total liver VOI)*Total liver clearance rate) and expressed as %/min/m^2^ [28]. Therefore, functional and volume evaluations of the three compartments were obtained at each time point.

### 2.5. Tumor Assessment

All patients had contrast-enhanced liver magnetic resonance imaging examination at 3 months after RE to evaluate tumor response upon mRECIST criteria.

### 2.6. Statistical Analysis

Qualitative and continuous variables were described using numbers, percentages, medians with interquartile ranges (IQR) or range, or means ± standard deviations (SD).

At each time point, the functions of the three compartments were compared with their baseline value using Wilcoxon signed-rank tests. Linear mixed models were used to take into account repeated measurements over time (at day 15, and 1, 2, 3, and 6 months after RE) in order to test the independence of the variations in volume and function from baseline both in the treated and nontreated livers. Patients were treated as random factors.

All statistical analyses were performed using the STATA software version 15.0 (Statacorp, College Station, TX, USA); two-tailed *p* values < 0.05 were considered significant.

## 3. Results

### 3.1. Population

**Figure 1:**
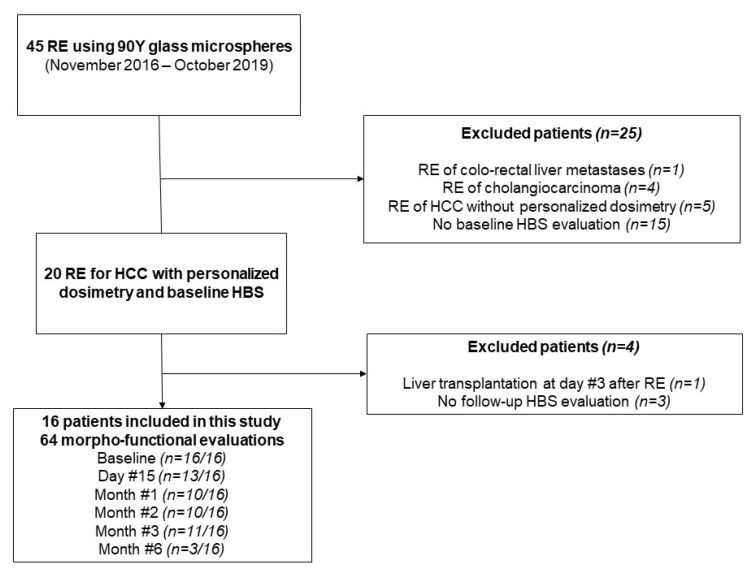
Study flow chart.

From November 2016 to October 2019, a total of 45 patients were treated by RE at our institution. Among them, 16 with baseline and follow-up morpho-functional imaging were included (see Flowchart, Figure 1). Patient and tumor characteristics are presented in Table 1. There were 15 patients who had cirrhosis, mainly from viral origin (63%), and classified as Child-Pugh A in all cases. Portal vein invasion was observed in 11 patients (69%). Although unilobar was in all patients, multifocal disease was noted in 11 patients (69%). The mean targeted tumor dose (based on the work-up phase) was 262 Gy (range: 205–461Gy). Regarding the 3 patients with a 6-month HBS examination, 1/3 had a right lobar portal vein invasion with an infiltrative tumor, whereas the others had multifocal disease with a median index tumor size of 73 mm.

A total of 64 morpho-functional examinations using HBS were performed over the study period. They were available at baseline for all patients with a median of one day (range: 1–14 days) before RE. During follow-up, they were obtained at 2 weeks (*n* = 11/16), 1 month (*n* = 13/16), 2 months (*n* = 10/16), and 3 months (*n* = 11/16) after RE. An additional 6-month examination was obtained in 3/16 patients in the context of a preoperative work-up before surgery. Over the study period, patients had a median of 4.5 morpho-functional evaluations (range: 2–6). Missing evaluations were due to mebrofenin shortage (*n* = 16), patient refusal (*n* = 2), or technical failure of the SPECT/CT system (*n* = 1).

### 3.2. Tumor Assessment

At 3 months, 31% (*n* = 5/16) had a complete response, 19% (*n* = 3/16) had a partial response, 25% (*n* = 4/16) had stable disease, and 25% (*n* = 4/16) had progressive disease. Tumor progression occurred in the nontreated livers in all cases. All 3 patients with a 6-month evaluation had tumor control (complete response (*n* = 1), partial response (*n* = 1), stable disease (*n* = 1)). Two of them underwent liver surgery, whereas the third one, with a complete response, was contraindicated because of severe pulmonary hypertension; he has been under surveillance without any tumor progression. To date (May 2021), 13/16 patients died, determining a median overall survival of 18.5 months after RE (range: 3–30). Three patients are still alive with 21, 17, and 17 months of overall survival after RE.

### 3.3. Function and Volume Changes

The median baseline functions of the whole, treated, and nontreated livers were 6.5%/min/m^2^ (IQR = 5.5–8.7), 2.1%/min/m^2^ (IQR = 1.6–3.6), and 4.0%/min/m^2^ (IQR = 3.2–5.5), respectively. The median baseline volumes of the treated livers and nontreated livers were 946 mL (IQR: 716–1130) and 975 mL (IQR: 819–1266), respectively. Their variations over time are presented in Table 2.

(1) Whole liver

**Figure 2:**
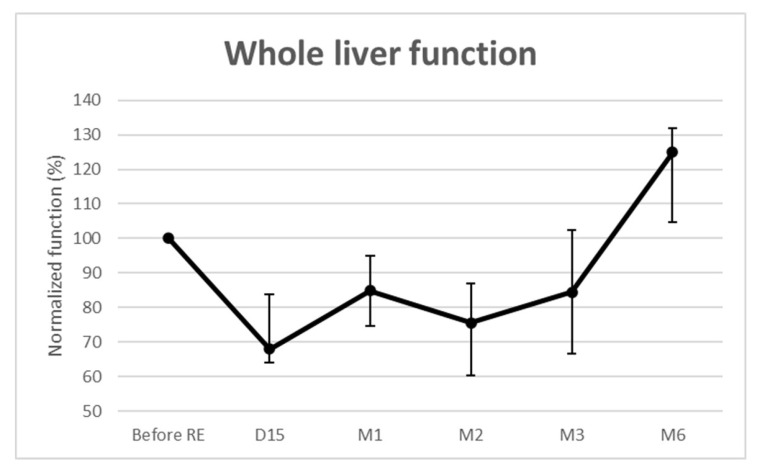
Functional changes from baseline of the whole livers.

Whole liver function significantly decreased over time (Figure 2). The median loss was maximal at 2 weeks (−32% (IQR: −36–−16.2%; *p* = 0.002)) and remained below baseline until month 3. It was then −15% (*p* = 0.002) at 1 month, −25% (*p* < 0.001) at 2 months, and −16% (*p* = 0.027) at 3 months. For the 3 patients evaluated at 6 months, the median whole liver function increased by 25% (IQR: 4.8–31.9%; *p* = 0.159). No REILD [10] was observed.

(2) Functional and volume changes in the treated livers

**Figure 3:**
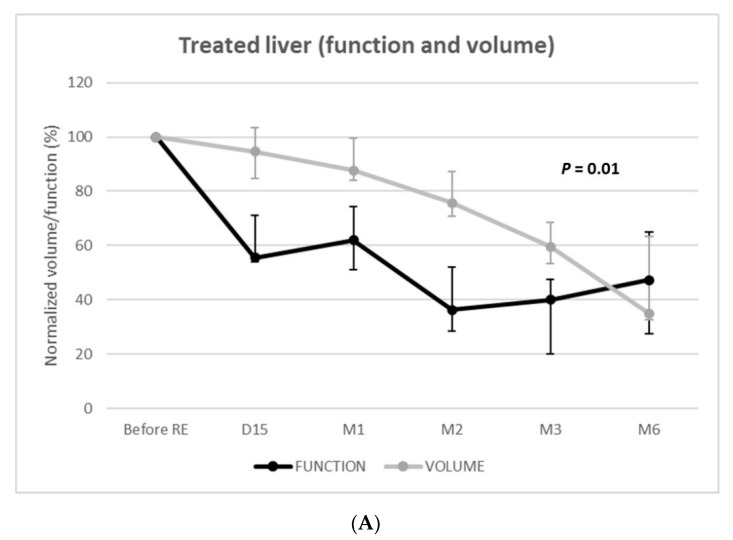

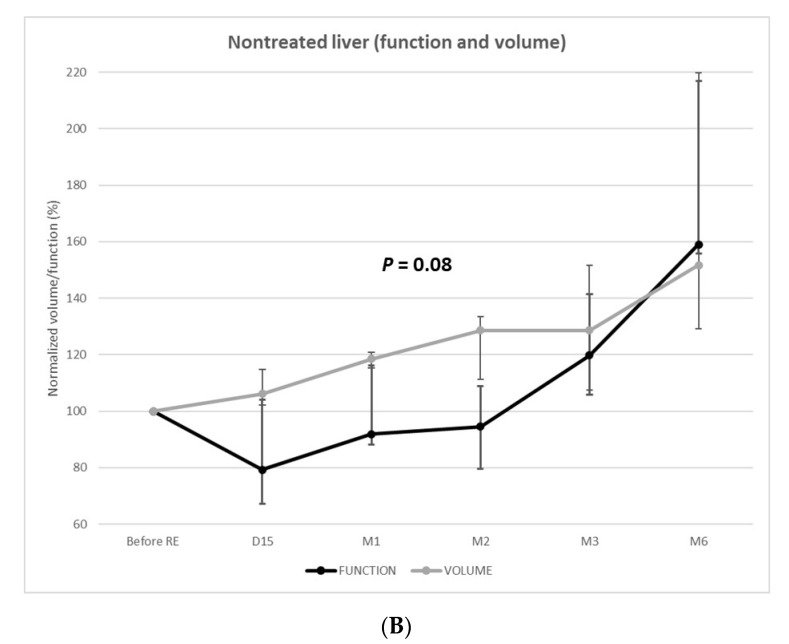
Function and volume changes from baseline of the treated (**A**) and nontreated (**B**) livers.

Function of the treated livers rapidly and strongly decreased to reach −64% (IQR = −80–−59%; *p* < 0.001) at 2 months and −60% (IQR = −80–−52%; *p* < 0.001) at 3 months (Figure 3). Volumetric and functional changes exhibited parallel (i.e., dependent) evolutions (*p* = 0.01) with linear mixed models. Nevertheless, volumetric loss underestimated functional loss until 6 months post-RE.

### 3.4. Comparison Using Linear Mixed Models

(3) Functional and volume changes in nontreated livers

The function of the nontreated livers unexpectedly decreased at 2 weeks (median =−21% [IQR: −33–4%]; *p* = 0.027). Among the 11 patients evaluated at 2 weeks, 8 (73%) presented nontreated liver function decrease (median: −26%) (Figure 3).

The decrease of function was −8% ((IQR = −12–16%), *p* = 0.249) and −6% ((IQR = −20–9%), *p* = 0.336) at 1 and 2 months, respectively. The function of the nontreated livers remained below the baseline level until 3 months post-RE, when a significant increase in function was observed (+20% (IQR: 6–41%); *p* = 0.002). At 6 months, the function of the nontreated livers significantly increased (+59% (IQR: 56–117%); *p* < 0.001) in the 3 patients, respectively, +53% (3.9 to 6.0%/min/m^2^), +59% (3.9 to 6.2%/min/m^2^), and +175% (1.8 to 4.9%/min/m^2^).

Over time, the volume and function of the nontreated livers varied independently (*p* = 0.08). The volume progressively increased, whereas function remained below the baseline level until 3 months post-RE.

## 4. Discussion

This study was the first to explore, through sequential HBS assessments, regional liver function changes following 90Y RE using personalized dosimetry. Some of the results were unexpected, such as the early decrease in function of the nontreated livers. The literature on the role of HBS in radioembolization is scarce. Two case studies (2–3 patients) [17,18] reported on the feasibility of HBS to monitor regional liver function changes after RE. Three larger case studies (13–35 patients) were recently published [19,20,21], but they were based on a single post-RE assessment (at 6 weeks [19], 2 months [21], or 3 months [20]), whereas this study analyzed liver function over time using serial measurements. Additionally, none of the previous reports used personalized dosimetry [6], which is destined to become the standard of care in the setting of ^90^Y RE [9].

Van der Velden et al. [20] showed that the function of treated livers declined at 3 months after RE, while the function of nontreated livers increased. Our results corroborate their single post-RE evaluation: −19% for whole liver function at 3 months versus −16% in our study; −52% for the treated liver function versus −60% in our study. At 3 months, the gain in the nontreated liver was lower in our study, with +20% (vs. +50% in [20]) but only 46% of their patients presented cirrhosis against 94% in our series, and regeneration is known to be slower and more limited in cirrhotic livers [22].

In 2020, Willowson et al. [21] investigated changes in liver function at 2 months using HBS after RE in 35 patients. They observed that only whole-liver treatment resulted in a significant decrease in global liver function, suggesting that function may shift from irradiated to spared zones. This is in contradiction to our results and can be attributed to considerable differences in patient selection and treatment modalities: only 1/35 patient had cirrhosis in their study versus 15/16 in ours; they used resin, whereas we used glass ^90^Y microspheres; most patients (26/35) underwent whole liver RE, whereas we kept a >30% hepatic reserve (i.e., non-treated liver volume) in all patients to preserve enough non-tumoral liver in accordance with the personalized dosimetry concept [9]. In our study, serial measurements demonstrated a significant loss (−15% to −25%) of whole liver function during the first 3 months after unilobar RE using personalized dosimetry. In the study by Labeur et al. [19], whole liver function significantly declined by 22% (*p* < 0.001) at 6 weeks after lobar RE, which matched our results.

The decrease in whole liver function corroborates the definition of REILD, which must be considered in any patient developing liver failure between 4 and 8 weeks after RE, excluding tumor growth or biliary obstruction [10,29]. Radiotherapy-induced liver disease (RILD) has been described as early as 2 weeks and may be observed until 7 months after external beam radiation therapy [30,31]. Our work shows that global functional damage occurs between 2 weeks and 3 months, suggesting that the current definition of REILD might be too restricted in terms of the time frame.

The 15–25% loss in global liver function after RE is explained not only by the deeply decreased function in the treated livers due to ^90^Y microspheres in non-tumoral livers but also by an unexpected decrease in the function of the nontreated liver as early as 2 weeks after RE (−21%). Among the 8/11 patients with a functional decrease in the nontreated livers, the deepest decrease even reached −32%. Whether radiobiology in the liver is well-described, with the activation and proliferation of hepatic stellate cells and the myofibroblastic transformation preceding the production of an extracellular matrix and hepatic fibrosis [32], the cause of the initial decrease in the function of the nontreated livers remains unknown. A direct effect of ^90^Y can be definitely ruled out because of its very limited course within tissues, as testified on post-RE ^90^Y PET/CT. We can hypothesize that such contralateral damage results from a cytokine and/or immune-mediated mechanism. A recent study showed that RE was followed by a systemic inflammatory response, induces early oxidative stress, and activates pro-inflammatory pathways leading to endothelial injury with an activation of the coagulation cascade [33]. Elucidating the mechanism of contralateral damage is beyond the scope of this work but is worth exploring because it opens the gate towards adjunctive therapies to protect non-irradiated livers and ultimately to better optimize RE.

RE offers the possibility not only to control or downstage tumors but also to trigger compensatory hypertrophy of an untreated liver to enlarge it as a future liver remnant (FLR) for subsequent surgical resection [22,24]. Liver atrophy–hypertrophy complex is induced by the regenerative response following radiation-induced hepatocyte injury [22]. In patients treated by unilobar glass-^90^Y RE for HCC, Palard et al. [23] reported similar volume atrophy of the treated lobe. The −60% decrease in treated liver function at 3 months also matches a previous series on HBS following RE [18,20]. Although a functional decrease of the treated liver was parallel to that of the volume (*p* = 0.01), the volume strongly underestimated the function; at day 15, the function of the treated liver had already decreased by −45%, whereas the decrease in volume was only −5%.

What occurs in the nontreated liver is of high importance in the context of downstaging/bridging to resection. Nontreated liver volume steadily increased from day 15 to month 6, in line with previous reports [23,34]. However, corresponding changes in liver function were opposed, with a significant decrease at day 15 and until 2–3 months after RE. Such volume/function discrepancy has already been reported in other liver regeneration strategies; while the volume underestimates the function of the FLR after portal vein embolization (PVE) [35], it is the opposite after surgery by associating liver partition and portal vein ligation (ALPPS) [36]. Unfortunately, HBS is still not widespread, meaning that surgical decisions are most frequently based solely on volumetric evaluations. Volume/function discrepancy after RE presents the same way as after ALPPS; during the first 3 months following RE, the FLR (i.e., non-treated liver) volume overestimates its function, which could lead to unsafe liver resection caused by insufficient FLR function.

The optimal time from ^90^Y treatment to resection remains debated [22,23]. Given our results, waiting at least 3 months after RE would be advisable to restore baseline whole liver function and take a benefit of contralateral liver regeneration. RE and PVE, as liver regenerative strategies, have often been opposed [19,37]. Among the drawbacks of RE, a much longer time necessary to increase the FLR and a modest FLR (volume) hypertrophy have been underlined [20] and confirmed in this series. Nevertheless, the +59% FLR functional gain at 6 months after RE is comparable to what has been reported with liver venous deprivation, the most efficient regenerative strategy to date [27,37]. Disease control provided by Y90 treatment, the “test-of-time” to ensure a durable tumor response and a lack of cancer progression, together with promising FLR functional gain, makes RE a very attractive method of downstaging/bridging to resection.

Several limitations to this study must be acknowledged, including its small sample size and retrospective design. Moreover, what happens before 15 days and after 6 months following RE remains to be investigated. Finally, because of the small sample size, we could neither perform any subgroup analysis nor explore the relationship between tissue-absorbed dose and regional liver function due to methodological considerations. However, the preliminary results we obtained in this study allowed us to define the time-points of interest for an upcoming multicentric prospective study with the aim to ultimately refine RE therapy.

## 5. Conclusions

RE of HCC using personalized dosimetry induces significant regional changes in liver function over time. As early as 15 days following RE, both the treated and nontreated liver showed a decreased function. Nontreated liver function recovered after 3 months and greatly increased afterwards. Repeated HBS using ^99m^Tc-mebrofenin opens the gate towards a better understanding of radiobiological effects in the liver to better personalize RE.

## Figures and Tables

**Table 1:** Characteristics of patients, tumors, and treatments.

Variable	Number (%) or Median (Range)
**Age (years)**	60 (46–83)
**Gender**	
Male	14 (88%)
Female	2 (12%)
**Underlying liver disease**	
Alcohol	3 (19%)
Hepatitis C	10 (63%)
Nonalcoholic steatohepatitis	2 (12%)
Noncirrhotic	1 (6%)
**Child-Pugh score**	
A5	15 (94%)
A6	1 (6%)
**Performance status**	
0	11 (69%)
1	5 (31%)
**BCLC classification**	
B	2 (12%)
C	14 (88%)
**Tumor distribution**	
Multifocal	11 (69%)
Unilateral	16 (100%)
**Tumor size**	
Unidimensional (mm)	71 (37–155)
Volume (cm^3^)	340 (32–1173)
**Prior therapy**	
Chemoembolization	6 (38%)
Resection/Ablation	2 (12%)
Sorafenib	2 (12%)
Combined treatments	2 (12%)
None	7 (44%)
**Alpha-foetoprotein**	
Normal, <20 ng/mL	5 (31%)
20–200 ng/mL	8 (50%)
≥200 ng/mL	3 (19%)
**Portal vein invasion**	
Yes	11 (69%)
Main	2 (18%)
Left/right branch	6 (54%)
Segmental	3 (27%)
**Treatment**	
Lobar right	12 (75%)
Lobar left	2 (12%)
Right sector (ant or post)	2 (12%)
**Dose *(Gy)***	
Tumor	262 (205–461)
Irradiated non-tumoral liver	112 (55–182)
Whole non-tumoral liver	53 (20–85)

Abbreviations: BCLC, Barcelona Clinic Liver Cancer; Absorbed dose determined on MAA SPECT/CT.

**Table 2:** Regional liver function and volume, relative to baseline.

	Day #15 (*n* = 11/16)	Month #1 (*n* = 13/16)	Month #2 (*n* = 10/16)	Month #3 (*n* = 11/16)	Month #6 (*n* = 3/16)
**Whole liver function** **(%)**	68.0 (64.0–83.8)	84.9 (74.7–94.9)	75.5 (60.3–86.9)	84.4 (66.7–102.3)	125.0 (104.8–131.9)
**Treated liver function** **(%)**	55.6 (53.9–71.1)	62.0 (51.1–74.4)	36.3 (28.4–52.0)	40.1 (20.2–47.5)	47.2 (27.6–64.9)
**Nontreated liver function** **(%)**	79.2 (67.1–104.0)	91.9 (88.1–116.3)	94.5 (79.6–108.8)	119.7 (105.9–141.3)	159.0 (155.9–216.8)
**Treated liver volume** **(%)**	95 (85–103)	88 (84–99)	76 (71–87)	60 (53–69)	35 (33–63)
**Nontreated liver volume** **(%)**	106 (102–115)	118 (115–121)	129 (111–133)	129 (107–152)	152 (129–222)

Baseline values equal 100% (e.g., 125% means an increase of 25% as compared to the baseline value). Median (IQR) values.

## Data Availability

The datasets used and/or analyzed during the current study are available from the corresponding author on reasonable request.
